# Molecular identification of the prey range of the invasive Asian paper wasp

**DOI:** 10.1002/ece3.826

**Published:** 2013-10-09

**Authors:** Darren F Ward, Ana Ramón-Laca

**Affiliations:** Landcare ResearchPrivate Bag 92170, Auckland, New Zealand

**Keywords:** Asian paper wasp, diagnostics, diet, *Polistes*, predator

## Abstract

The prey range of the invasive Asian paper wasp, *Polistes chinensis antennalis*, was studied using molecular diagnostics. Nests of paper wasps were collected from urban residential and salt marsh habitats, larvae were removed and dissected, and DNA in the gut of the paper wasp larvae was amplified and sequenced with cytochrome c oxidase subunit I (COI). Seventy percent of samples (211/299) yielded medium-to high-quality sequences, and prey identification was achieved using BLAST searches in BOLD. A total of 42 taxa were identified from 211 samples. Lepidoptera were the majority of prey, with 39 taxa from 91% of samples. Diptera was a relatively small component of prey (three taxa, 19 samples). Conclusive species-level identification of prey was possible for 67% of samples, and genus-level identification, for another 12% of samples. The composition of prey taken was different between the two habitats, with 2.5× more native prey species being taken in salt marsh compared with urban habitats. The results greatly extend the prey range of this invasive species. The technique is a more effective and efficient approach than relying on the collection of “prey balls”, or morphological identification of prey, for the study of paper wasps.

## Introduction

Paper wasps (*Polistes*) are widely distributed around most of the globe and are diverse and common in many landscapes (Reeve 1991; Carpenter 1996). They are a very well-known model organism for the evolution of eusociality and behavior (Turillazi 1996; Starks and Turillazzi 2006). Despite this, little is known about the specifics of their foraging behavior; the range of prey taken; and how these may change seasonally, spatially, or with competition (Kasper et al. 2004; Brown et al. 2012). Paper wasps are likely to influence many other species in terrestrial ecosystems because they are voracious predators of invertebrates (Richter 2000; Nannoni et al. 2001; Kumar et al. 2009).

**Figure 1:**
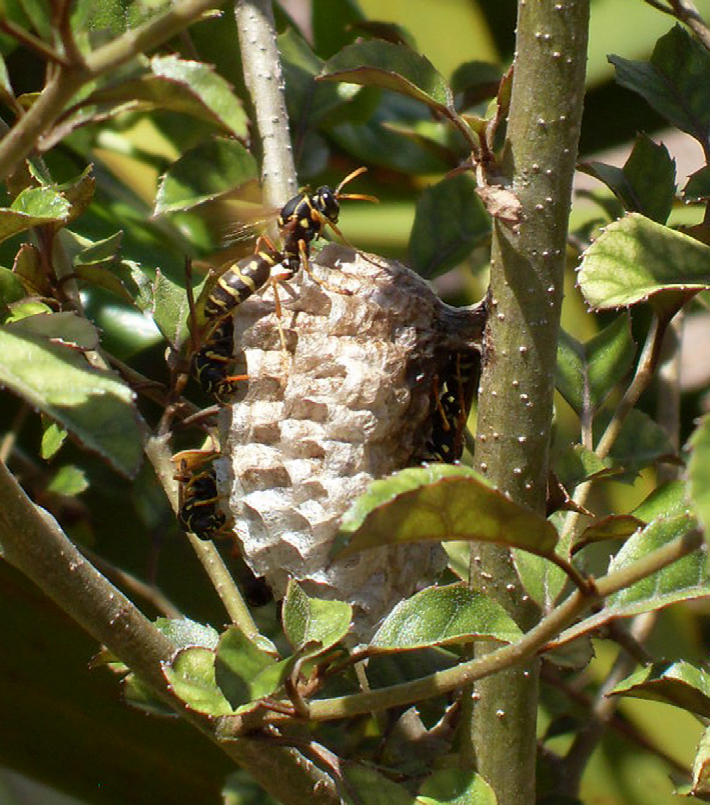
Asian paper wasps, *Polistes chinensis antennalis*, and nest.

Several species of paper wasps have also become invasive around the world: *P. versicolor* (Olivier) in the Galápagos, *P. dominula* in North America, and *P. humilis* (Fabricius) and *P. chinensis antennalis* Pérez both in New Zealand (Beggs et al. 2011). One hypothesis for the successful invasion of *P. dominula* in the USA is the broad range of prey it utilizes (Cervo et al. 2000). In New Zealand, the Asian paper wasp (*P. chinensis antennalis*) can reach densities of up to 210 nests/ha, translating into ∼1000–2000 wasps/ha, and many 10,000 of prey captured per season (Clapperton 1999; Fig. 1). Consequently, there are concerns that Asian paper wasps could have a significant impact on the native invertebrate fauna in native habitats (Clapperton 1999). Thus, it is important to know the details of a species' foraging behavior and its precise prey range to better understand the role of paper wasps in native ecosystems and the impacts of invasive paper wasp species.

Traditionally, prey items of social wasps are collected from foraging wasps as they return to the nest in the form of a “pellet” or “prey ball” (a masticated partial remain of a prey carried by the worker wasp) and identified (Harris 1991; Gambino 1992; Harris and Oliver 1993; Clapperton 1999). However, the identification based on the morphological features of prey from highly masticated prey balls is likely to affect accurate identification and also underestimate the range of prey. For example, Gambino (1992) could only identify ∼17% of prey items using morphology and suggested that this may favor features that are more easily observed, thus influencing identifications and conclusions. This was supported by Kasper et al. (2004) who found only 39% of *P. humilis* prey could be identified using morphological features.

Molecular identification of the prey of social wasps has been examined recently by Wilson et al. (2009) who examined the prey of the yellowjacket, *Vespula pensylvanica*. Molecular identification of prey balls showed the wide breadth of prey consumed and helped quantify the direct and indirect effects on native ecosystems in Hawaii. However, while the collection and analyses of prey balls are suitable for *Vespula* wasps (because of their large nests and high foraging rates), the collection of prey balls is extremely time-consuming for paper wasps, which have much smaller colonies.

In this paper, we utilize molecular diagnostics to examine prey range of the Asian paper in New Zealand wasp and compare the prey taken from two different habitats.

## Materials and Methods

### Collection of samples

Nests were collected from two habitats in the austral summer of 2012 and 2013 (Table 1). In urban habitats, nests were collected from wooden fences, residential gardens, and plantings of native shrubs, from six sites in Auckland (suburbs of St Johns and Glenn Innes; −36.878, 174.838) and from one site in central Whangarei (−35.721, 174.314). In salt marshland habitats, nests were collected from short (<2 m high) plants (typically *Coprosma* and *Corokia*) from five sites in Auckland city: Tahuna Torea Reserve (−36.871, 174.883), Devonport (−36.817, 174.794), Hillary Street reserve (−36.804, 174.780), Tuft Crater (−36.802, 174.749), O'Neill's Cemetery (−36.810, 174.780); and two sites in the wider Auckland region: Huia (−36.997, 174.567) and Omaha (−36.342, 174.772).

**Table 1:** The number of sites, nests collected, and larvae submitted for sequencing

	2012			2013			Total		
			
Habitat	Sites	Nests	Larvae	Sites	Nests	Larvae	Sites	Nests	Larvae
Urban	6	17	66	4	9	43	7	26	109
Salt Marsh	4	11	43	4	28	147	7	39	190
Total	10	28	109	8	37	190	14	65	299

**Figure 2:**
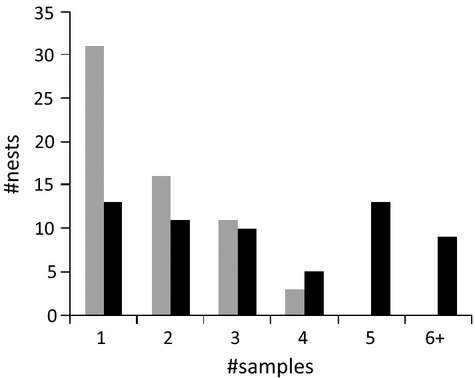
The number of larvae submitted for sequencing (black bars) and the resulting number of prey identified (gray bars) from a paper wasp nest.

Nests of the Asian paper wasp were discovered by walking slowly along walking tracks and through urban and salt marsh habitats. Nests were sprayed with household fly spray to disperse adult wasps, while the nest was removed and placed in a plastic bag. Bags were subsequently kept at −20°C until the larvae were removed from the nest with forceps, and their “gut” was dissected for molecular analysis. Nests ranged in size from 68 to 186 cells (4–8 cm diameter). The number of larvae removed from a nest largely depended on the size of the nest, but the range of larvae removed was from 1 to 6 (Fig. 2). In most cases, a maximum of six larvae were removed from a single nest, and larvae were taken from around the sides rather than from just one section of the nest. For two nests, we removed larger numbers of larvae (*n* = 9, 17) in order to examine raising sample sizes on the number of species identified. Not all larvae were removed from nests and sequenced because of financial constraints. In total, 299 larvae were sequenced, but the total number of larvae in all the nests was >500.

### DNA extraction, amplification, and sequencing

DNA was extracted from the gut of the paper wasp larvae. Samples were digested in 500 μL of DXT buffer (Qiagen®, Hilden, Germany) and 5 μL of proteinase K and incubated overnight at 56°C. DNA was then extracted in an automated extraction machine (QIAxtractor®, Hilden, Germany) following manufacturer's instructions. DNA was eluted in 70 μL of DXE (Qiagen®) and then stored at 4°C for immediate use. Selective polymerase chain reaction (PCR) amplifications of a short region of 307 bp of the *Cytochrome c oxidase subunit I* (COI) was employed to target the gut content because the primers used have low affinity with Hymenoptera (Rougerie et al. 2011). PCR amplification was carried out in 15-μL reactions containing 1 × PCR buffer (with 2 mmol/L MgCl_2_), 0.2 mmol/L dNTP and 1 U of FastStart Taq DNA Polymerase (Roche Diagnostics), 0.4 μL of 0.01% BSA, and primers LepF1 (5′-ATTCAACCAATCATAAAGATATTGG-3′) and MLepR1 (formerly named MH-MR1) (5′-CCTGTTCCAGCTCCATTTTC-3′) (Hajibabaei et al. 2006) with 5 μL of DNA. Thermocycling conditions were 5 min of initial denaturation at 94°C; a touchdown of 10 cycles of 40 sec at 94°C, 40 sec at an annealing temperature decreasing 0.5°C every cycle from 55°C to 50°C, and 1 min at 72°C, followed by 38 more cycles of 40 sec at 94°C, 40 sec at 50°C, 1 min at 72°C; and a final extension of 5 min at 72°C. PCR products were sequenced directly using a BigDye Terminator v3.1 Cycle Sequencing Kit (Applied Biosystems®, Foster City, CA) and then analyzed on an ABI310 capillary sequencer. Resulting sequences were edited using Geneious R.6 created by Biomatters (http://www.geneious.com).

### DNA identification of prey

Identification was achieved using the Barcode of Life Data Systems (BOLD) identification engine to search the full DNA barcode records in BOLD for a match. In all cases, the probability of placement (%) at a given taxonomic level was recorded. When a species-level match was unavailable, the nearest match was recorded. However, we cross-checked all suggested matches with the most recent taxonomic index for New Zealand (Gordon 2010) and especially for Lepidoptera (Hoare 2010), which made up the majority of records. We took a conservative approach and did not list a prey item to species, or genus, level unless a high BOLD match and the taxa were listed in Hoare (2010).

## Results

### DNA amplification and sequencing

Two hundred and ninety-nine samples were submitted for molecular analysis, from which 70% yielded medium-to high-quality sequences used for prey identification. There was some repetition of prey taxa recorded from the same nest; however, multiple prey species (>1 taxa) were present in 50% of nests (Fig. 2). The average number of prey taxa identified from a single paper wasp nest was 1.8, with a maximum number of four prey taxa identified from a single nest. However, this is dependent on the number of larval sequenced from each nest, which was not the same for each nest. In general, the number of prey taxa identified from a single paper wasp nest was significantly lower (χ^2^ = 50.09, df = 5, *P* < 0.001) than the number of larval samples sequenced from a nest, because of repetition of the same prey species.

### Identification of prey

A total of 42 taxa were identified from 211 samples (Table 2; Fig. S1). Lepidoptera (butterflies, moths) were the majority of identifications, with 39 taxa (93% of total) and 192 samples (91% of total). Diptera (flies) was a relatively small component (three taxa, 19 samples) of prey. Conclusive species-level identification of prey was possible for 67% of samples, and genus-level identification was obtained for another 12% of samples (Table 2). Matches to species level, or genus level, could not be confidently obtained for 20% of samples, including all Diptera and some taxa from three of the largest subfamilies of Lepidoptera (Geometridae, Noctuidae, Tortricidae).

**Table 2:** Identification of prey from the Asian paper wasp using DNA sequences

Identification	No Samples	% Match with BOLD	GenBank Accession
Lepidoptera			
Coleophoridae			
*Coleophora*^1^,^2^	1	94.23	KF153714
Crambidae			
*Uresiphita ornithopteralis*^2^	6	99.33–99.66	KF153894–KF153899
Elachistidae			
*Elachista*^1^	1	97.31	KF153736
Geometridae			
*Anachloris subochraria*	4	99.19–99.33	KF153702–KF153705
*Chloroclystis*^1^,^2^	7	98.94–100	KF153706–KF153712
*Declana leptomera*	1	100	KF153722
Geometridae 1^3^	2	95.24–95.29	KF153746–KF153747
Geometridae 2^3^	1	95.29	KF153741
Geometridae 3^3^	3	95.62–96.85	KF153743–KF153745
Geometridae 4^3^	1	96.82	KF153742
*Microdes*^1^	3	94.87	KF153776–KF153778
*Phrissogonus laticostata*^2^	10	100	KF153818–KF153827
*Poecilasthena pulchraria*	14	97.94–99.32	KF153828–KF153841
*Scopula rubraria*	9	98.99–100	KF153846–KF153854
Lycaenidae			
*Lampides boeticus*^2^	21	99.66–100	KF153750–KF153770
*Lycaena*	1	96.62	KF153771
*Lycaena salustius*	3	98.65	KF153772–KF153774
*Zizina labradus*	1	99.16	KF153912
Noctuidae			
*Chrysodeixis eriosoma*	1	100	KF153713
*Ctenoplusia albostriata*^2^	4	100	KF153715–KF153718
*Ctenoplusia limbirena*^2^	2	100	KF153719–KF153720
*Ectopatria aspera*^4^	13	98.64–100	KF153723–KF153735
*Helicoverpa armigera*^2^	2	99.66–100	KF153748–KF153749
*Mythimna separata*^2^	33	98.35–100	KF153779–KF153811
Noctuidae 1^3^	1	96.63	KF153813
Noctuidae 2^3^	1	96.41	KF153816
Noctuidae 3^3^	4	100	KF153812, KF153814–KF153815, KF153817
*Spodoptera litura*^2^	4	99.19–100	KF153855–KF153858
*Thysanoplusia orichalcea*^2^	5	100	KF153867–KF153871
Nymphalidae			
*Danaus plexippus*^2^	1	100	KF153721
Oecophoridae			
*Tachystola*^1^	1	95.31	KF153866
Pyralidae			
*Ptyomaxia trigonogramma*^2^	4	99.66–100	KF153842–KF153845
*Vinicia*	12	97.61–98.61	KF153900–KF153911
Tortricidae			
*Epiphyas postvittana*^2^	4	99.63–100	KF153737–KF153740
*Merophyas divulsana*^2^	1	100	KF153775
Tortricidae 1^3^	3	93.33–96.86	KF153891–KF153893
Tortricidae 2^3^	1	95.31	KF153890
Tortricidae 3^3^	1	95.98	KF153888
Tortricidae 4^3^	5	93.94–96.86	KF153884–KF153887, KF153889
Diptera			
Syrphidae^3^	6	94.36–97.16	KF153859–KF153864
Tachinidae^3^	1	94.38	KF153865
Tipulidae^3^	12	99.34–100	KF153872–KF153883

1 BLAST did not identify the taxa, or the closest species-level match is not listed from New Zealand (Hoare 2010).

2 Introduced species.

3 No match or low percentage match at genus or species level.

4 Hoare (2010) used to confirm species.

Of the Lepidoptera, 24 species (62%) are endemic or native, and fifteen species (38%) are introduced species in New Zealand. None of the endemic species are threatened (Stringer et al. 2012). However, several of the introduced species are considered pests, and some of them are economically important (Tooman et al. 2011).

### Comparison of habitats

**Figure 3:**
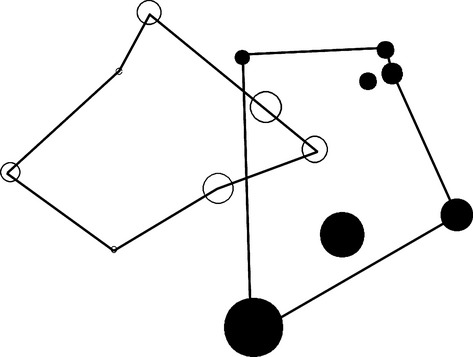
Nonmetric MDS ordination plot comparing the composition of prey species from urban (open circles) and salt marsh (black circles) habitats. The size of the circle indicates a greater proportion of native and endemic prey species at that site.

There was a significant difference between urban and salt marsh habitats in the composition of prey species caught by paper wasps (ANOSIM R value = 0.294, *P* = 0.01, Fig. 3). There was a significantly higher proportion (odds ratio 2.5×) of native species preyed upon in the salt marsh habitat compared with urban habitat (2 × 2 contingency table, χ^2^ = 10.08, df = 1, *P* < 0.05). Common species prey upon were *Mythimna separata* (Cosmopolitan Armyworm) and *Lampides boeticus* (Pea blue butterfly), both introduced, and common native species were *Poecilasthena pulchraria* and *Ectopatria aspera* (Table 2).

## Discussion

Molecular diagnostics of prey items show that the Asian paper wasp is a generalist predator on a very wide range of prey. Asian paper wasps consumed a number of introduced pest species, and some of them are economically important (e.g., *Helicoverpa armigera, Epiphyas postvittana*) and may have a beneficial role in some areas such as residential gardens or commercial crops. For example, Gould and Jeanne (1984) showed that by preying on *Pieris rapae* caterpillars, *Polistes* wasps improved the weight and quality of cabbages.

Our results also show that a number of native species were also predated upon. There is existing anecdotal evidence that Asian paper wasps have an impact on native species. Of most concern are impacts on already vulnerable species of native Lepidoptera. Although by consuming a wide range of prey, the impacts on a few specific species may be less severe. However, population level impacts have not yet been undertaken.

Molecular identification of the gut contents is becoming increasingly common for a wide range of invertebrates (Rougerie et al. 2011; Derocles et al. 2012; Gariepy et al. 2012), particularly for providing information that is time-consuming or difficult to obtain by conventional rearing or gut dissection, for example, host ranges of parasitoids (Rougerie et al. 2011; Derocles et al. 2012), or vertebrates hosts of tick species (Gariepy et al. 2012).

For wasps, molecular methods offer a significant advantage over identification based on morphology, where visual identifications are not fully representative of the types and proportions of prey taken (Kasper et al. 2004). The technique demonstrated here is more accurate than identifications based on morphology. In this study, 50% of prey species (21 from a total of 42 taxa) were identified to species and 67% to genus (28 of 42). This compares with only 20% species-level identification (45% to genus) with morphological identification for the same paper wasp species (Clapperton 1999).

However, identifications based on molecular methods depend largely on the availability and accuracy of sequences in molecular databases. We believe our percentage level of species identification could be greatly improved, but molecular databases are currently lacking many associated sequences/taxa from New Zealand. For example, only 33% (9/27) of the endemic or native taxa could conclusively be identified to species level using BOLD, but 87% (13/15) of introduced taxa could be identified to species. This notion is further supported by very limited extent of publically available records of “Lepidoptera” from New Zealand (150 sequences, 53 #BINs, 24 species; BOLD search 30/5/2013). Several such “gaps” in molecular databases have recently been explored (Virgilio et al. 2010; Kvist 2013).

Although our results are consistent with prey items previously found for paper wasps in New Zealand (Clapperton 1999), the molecular approach used in this study might have overlooked any Hymenoptera prey (especially bees) because the primers used have low affinity for this group. In addition, the occurrence of multiple prey species, as in Kasper et al. (2004), could have been missed. Other molecular techniques such as next-generation sequencing could be used to overcome DNA mixtures, but these methods can be time-consuming and highly expensive.

The technique of collecting paper wasp larvae from the nest is far more time-efficient than collecting prey balls from foraging workers. Clapperton (1999) found that, on average, an Asian paper wasp worker returns to the nest every 2–5 min, but only one-third of these have a prey balls food pellet (other foragers carry debris for nest building, liquid food, or return without anything). Thus, on average, only 4–10 prey balls could be collected per hour. Clapperton (1999) collected 103 prey balls from 27 nests over 1 month. For *P. humilis* in Australia, Kasper et al. (2004) collected 44 prey balls from 514 foragers (a return rate of 0.08 prey balls per wasp or 12 wasps needed for one prey balls). These data show that collecting food prey balls from foraging paper wasps as they return to the nest is a very time-consuming task. Conversely, the technique demonstrated here presents data for 211 samples from 62 nests, collected over 9 days (partial) in the field. Furthermore, not all nests were collected during these trips, some being left for additional studies. In addition, not all larvae were removed from nests and sequenced. In total, 299 larvae were sequenced (70% were successful), but the total number of larvae that could have been sequenced was >500.

Another significant advantage of using molecular identifications is that further taxonomic identifications can be made to sequences in the future (e.g., lower levels of taxonomic information can be added), or identifications can be changed. This is not possible for morphological identifications, particularly as samples are often discarded at the end of the study. Thus, the use of molecular identifications provides an ongoing resource and potentially a collaborative one (e.g., Smith et al. 2012), which can greatly improve knowledge of a particular species or diversity at specific locations.

In conclusion, molecular diagnostics have shown the Asian paper wasp to be a generalist predator on a very wide range of prey, possibly contributing to its success as an invasive species. This technique may open further avenues for studying the foraging behavior and diet of paper wasps, a neglected aspect of this relatively well-known group.

## Data Accessibility

GenBank accessions KF153702–KF153912 are listed in Table 2. All sequences and collection data are available on BOLD (http://www.boldsystems.org) to all BOLD users under the project “New Zealand Paper Wasp Prey (NZPWP)”.

## Supporting Information

**Figure S1.** Neighbor-joining tree based on Kimura 2-parameter genetic distances of 211 sequences of prey of the Asian paper wasp *P. chinensis antennalis*. Label information includes: taxonomic name, BOLD process ID, BOLD sample ID, and common name (where available).
