# Supplementary material for: Molecular identification of the prey range of the invasive Asian paper wasp
Source: Ecol Evol. 2013 Oct 9;3(13):4408–14. doi: 10.1002/ece3.826 (PMC3856741; doi:10.1002/ece3.826)
Supplement: Supplementary file 1 [file ece30003-4408-SD1.pdf]

BOLD TaxonID Tree

Title	:	New Zealand Paper Wasp Prey [NZPWP]
Date	:	29-May-2013
Data Type	:	Nucleotide
Distance Model	:	Kimura 2 Parameter
Marker	:	COI-5P
Codon Positions	:	1st, 2nd, 3rd
Labels	:	Extra Info, SampleID, ProcessID
Filters	:	Length > 200

Sequence Count	:	211
Species count	:	21
Genus count	:	26
Family count	:	13
Unidentified	:	68

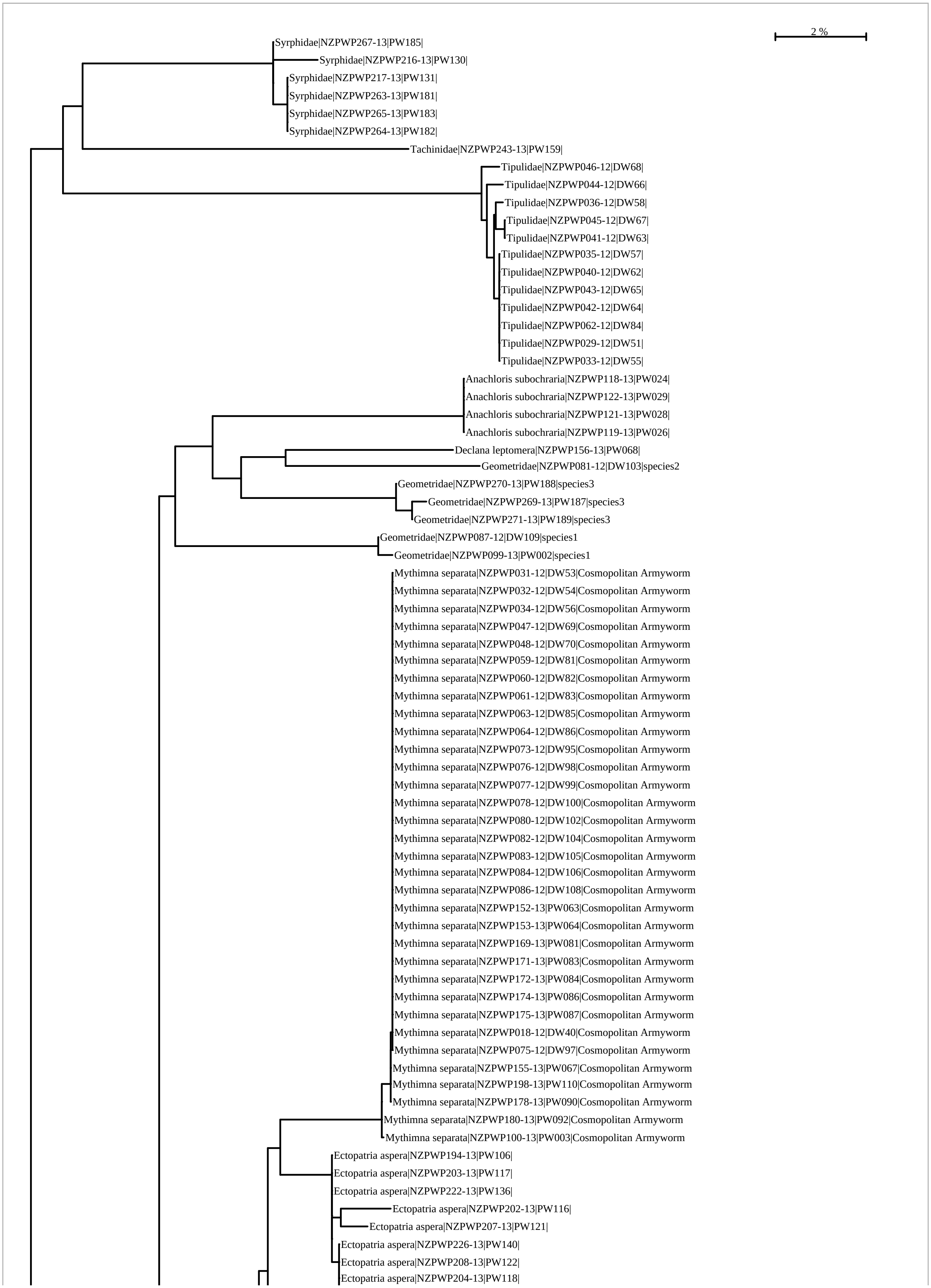
2 %
Syrphidae|NZPWP267-13|PW185|
Syrphidae|NZPWP216-13|PW130|
Syrphidae|NZPWP217-13|PW131|
Syrphidae|NZPWP263-13|PW181|
Syrphidae|NZPWP265-13|PW183|
Syrphidae|NZPWP264-13|PW182|
Tachinidae|NZPWP243-13|PW159|
Tipulidae|NZPWP046-12|DW68|
Tipulidae|NZPWP044-12|DW66|
Tipulidae|NZPWP036-12|DW58|
Tipulidae|NZPWP045-12|DW67|
Tipulidae|NZPWP041-12|DW63|
Tipulidae|NZPWP035-12|DW57|
Tipulidae|NZPWP040-12|DW62|
Tipulidae|NZPWP043-12|DW65|
Tipulidae|NZPWP042-12|DW64|
Tipulidae|NZPWP062-12|DW84|
Tipulidae|NZPWP029-12|DW51|
Tipulidae|NZPWP033-12|DW55|
Anachloris subochraria|NZPWP118-13|PW024|
Anachloris subochraria|NZPWP122-13|PW029|
Anachloris subochraria|NZPWP121-13|PW028|
Anachloris subochraria|NZPWP119-13|PW026|
Declana leptomera|NZPWP156-13|PW068|
Geometridae|NZPWP081-12|DW103|species2
Geometridae|NZPWP270-13|PW188|species3
Geometridae|NZPWP269-13|PW187|species3
Geometridae|NZPWP271-13|PW189|species3
Geometridae|NZPWP087-12|DW109|species1
Geometridae|NZPWP099-13|PW002|species1
Mythimna separata|NZPWP031-12|DW53|Cosmopolitan Armyworm
Mythimna separata|NZPWP032-12|DW54|Cosmopolitan Armyworm
Mythimna separata|NZPWP034-12|DW56|Cosmopolitan Armyworm
Mythimna separata|NZPWP047-12|DW69|Cosmopolitan Armyworm
Mythimna separata|NZPWP048-12|DW70|Cosmopolitan Armyworm
Mythimna separata|NZPWP059-12|DW81|Cosmopolitan Armyworm
Mythimna separata|NZPWP060-12|DW82|Cosmopolitan Armyworm
Mythimna separata|NZPWP061-12|DW83|Cosmopolitan Armyworm
Mythimna separata|NZPWP063-12|DW85|Cosmopolitan Armyworm
Mythimna separata|NZPWP064-12|DW86|Cosmopolitan Armyworm
Mythimna separata|NZPWP073-12|DW95|Cosmopolitan Armyworm
Mythimna separata|NZPWP076-12|DW98|Cosmopolitan Armyworm
Mythimna separata|NZPWP077-12|DW99|Cosmopolitan Armyworm
Mythimna separata|NZPWP078-12|DW100|Cosmopolitan Armyworm
Mythimna separata|NZPWP080-12|DW102|Cosmopolitan Armyworm
Mythimna separata|NZPWP082-12|DW104|Cosmopolitan Armyworm
Mythimna separata|NZPWP083-12|DW105|Cosmopolitan Armyworm
Mythimna separata|NZPWP084-12|DW106|Cosmopolitan Armyworm
Mythimna separata|NZPWP086-12|DW108|Cosmopolitan Armyworm
Mythimna separata|NZPWP152-13|PW063|Cosmopolitan Armyworm
Mythimna separata|NZPWP153-13|PW064|Cosmopolitan Armyworm
Mythimna separata|NZPWP169-13|PW081|Cosmopolitan Armyworm
Mythimna separata|NZPWP171-13|PW083|Cosmopolitan Armyworm
Mythimna separata|NZPWP172-13|PW084|Cosmopolitan Armyworm
Mythimna separata|NZPWP174-13|PW086|Cosmopolitan Armyworm
Mythimna separata|NZPWP175-13|PW087|Cosmopolitan Armyworm
Mythimna separata|NZPWP018-12|DW40|Cosmopolitan Armyworm
Mythimna separata|NZPWP075-12|DW97|Cosmopolitan Armyworm
Mythimna separata|NZPWP155-13|PW067|Cosmopolitan Armyworm
Mythimna separata|NZPWP198-13|PW110|Cosmopolitan Armyworm
Mythimna separata|NZPWP178-13|PW090|Cosmopolitan Armyworm
Mythimna separata|NZPWP180-13|PW092|Cosmopolitan Armyworm
Mythimna separata|NZPWP100-13|PW003|Cosmopolitan Armyworm
Ectopatria aspera|NZPWP194-13|PW106|
Ectopatria aspera|NZPWP203-13|PW117|
Ectopatria aspera|NZPWP222-13|PW136|
Ectopatria aspera|NZPWP202-13|PW116|
Ectopatria aspera|NZPWP207-13|PW121|
Ectopatria aspera|NZPWP226-13|PW140|
Ectopatria aspera|NZPWP208-13|PW122|
Ectopatria aspera|NZPWP204-13|PW118|

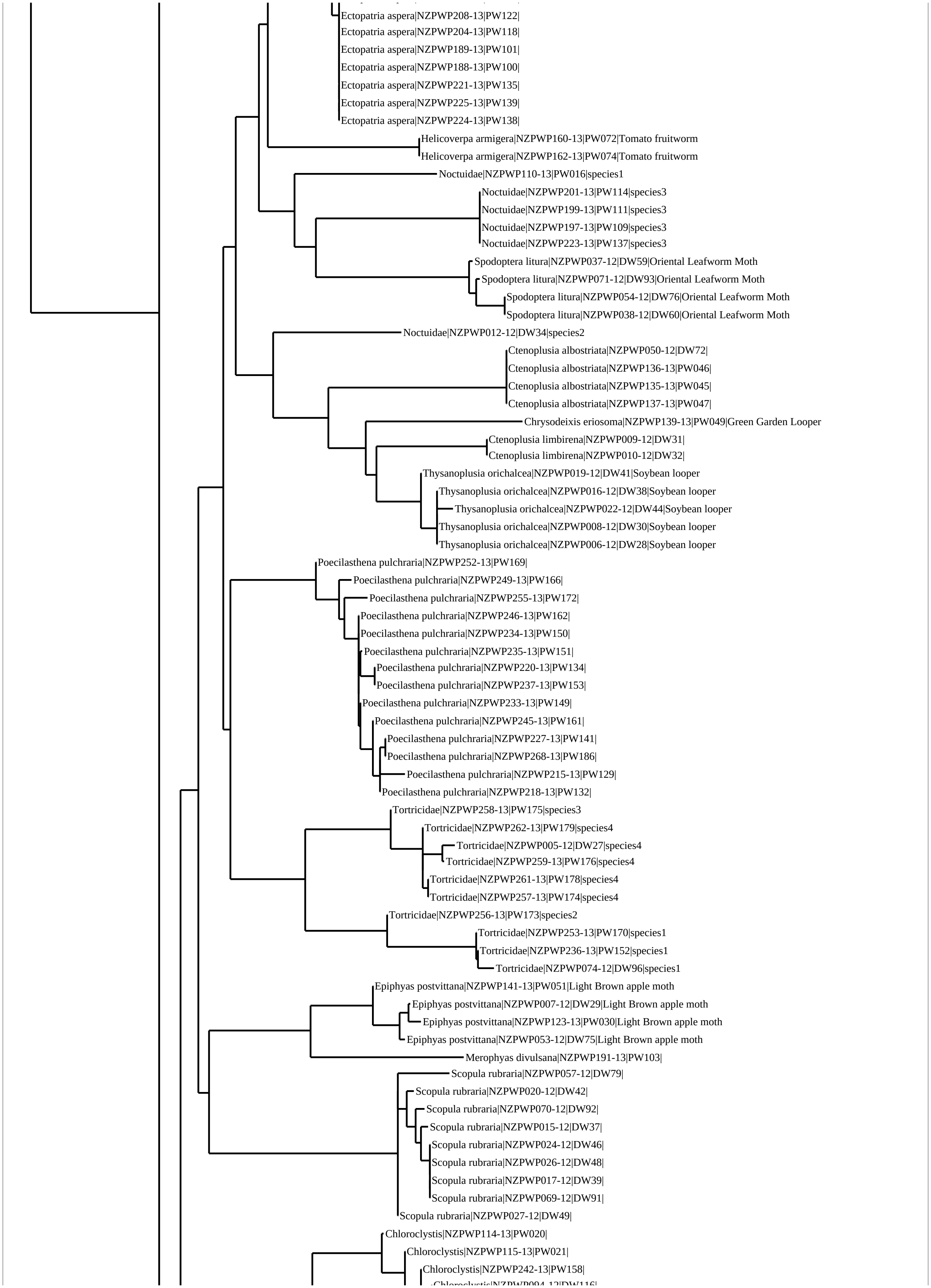
Ectopatria aspera|NZPWP208-13|PW122|
Ectopatria aspera|NZPWP204-13|PW118|
Ectopatria aspera|NZPWP189-13|PW101|
Ectopatria aspera|NZPWP188-13|PW100|
Ectopatria aspera|NZPWP221-13|PW135|
Ectopatria aspera|NZPWP225-13|PW139|
Ectopatria aspera|NZPWP224-13|PW138|
Helicoverpa armigera|NZPWP160-13|PW072|Tomato fruitworm
Helicoverpa armigera|NZPWP162-13|PW074|Tomato fruitworm
Noctuidae|NZPWP110-13|PW016|species1
Noctuidae|NZPWP201-13|PW114|species3
Noctuidae|NZPWP199-13|PW111|species3
Noctuidae|NZPWP197-13|PW109|species3
Noctuidae|NZPWP223-13|PW137|species3
Spodoptera litura|NZPWP037-12|DW59|Oriental Leafworm Moth
Spodoptera litura|NZPWP071-12|DW93|Oriental Leafworm Moth
Spodoptera litura|NZPWP054-12|DW76|Oriental Leafworm Moth
Spodoptera litura|NZPWP038-12|DW60|Oriental Leafworm Moth
Noctuidae|NZPWP012-12|DW34|species2
Ctenoplusia albostriata|NZPWP050-12|DW72|
Ctenoplusia albostriata|NZPWP136-13|PW046|
Ctenoplusia albostriata|NZPWP135-13|PW045|
Ctenoplusia albostriata|NZPWP137-13|PW047|
Chrysodeixis eriosoma|NZPWP139-13|PW049|Green Garden Looper
Ctenoplusia limbirena|NZPWP009-12|DW31|
Ctenoplusia limbirena|NZPWP010-12|DW32|
Thysanoplusia orichalcea|NZPWP019-12|DW41|Soybean looper
Thysanoplusia orichalcea|NZPWP016-12|DW38|Soybean looper
Thysanoplusia orichalcea|NZPWP022-12|DW44|Soybean looper
Thysanoplusia orichalcea|NZPWP008-12|DW30|Soybean looper
Thysanoplusia orichalcea|NZPWP006-12|DW28|Soybean looper
Poecilasthena pulchraria|NZPWP252-13|PW169|
Poecilasthena pulchraria|NZPWP249-13|PW166|
Poecilasthena pulchraria|NZPWP255-13|PW172|
Poecilasthena pulchraria|NZPWP246-13|PW162|
Poecilasthena pulchraria|NZPWP234-13|PW150|
Poecilasthena pulchraria|NZPWP235-13|PW151|
Poecilasthena pulchraria|NZPWP220-13|PW134|
Poecilasthena pulchraria|NZPWP237-13|PW153|
Poecilasthena pulchraria|NZPWP233-13|PW149|
Poecilasthena pulchraria|NZPWP245-13|PW161|
Poecilasthena pulchraria|NZPWP227-13|PW141|
Poecilasthena pulchraria|NZPWP268-13|PW186|
Poecilasthena pulchraria|NZPWP215-13|PW129|
Poecilasthena pulchraria|NZPWP218-13|PW132|
Tortricidae|NZPWP258-13|PW175|species3
Tortricidae|NZPWP262-13|PW179|species4
Tortricidae|NZPWP005-12|DW27|species4
Tortricidae|NZPWP259-13|PW176|species4
Tortricidae|NZPWP261-13|PW178|species4
Tortricidae|NZPWP257-13|PW174|species4
Tortricidae|NZPWP256-13|PW173|species2
Tortricidae|NZPWP253-13|PW170|species1
Tortricidae|NZPWP236-13|PW152|species1
Tortricidae|NZPWP074-12|DW96|species1
Epiphyas postvittana|NZPWP141-13|PW051|Light Brown apple moth
Epiphyas postvittana|NZPWP007-12|DW29|Light Brown apple moth
Epiphyas postvittana|NZPWP123-13|PW030|Light Brown apple moth
Epiphyas postvittana|NZPWP053-12|DW75|Light Brown apple moth
Merophyas divulsana|NZPWP191-13|PW103|
Scopula rubraria|NZPWP057-12|DW79|
Scopula rubraria|NZPWP020-12|DW42|
Scopula rubraria|NZPWP070-12|DW92|
Scopula rubraria|NZPWP015-12|DW37|
Scopula rubraria|NZPWP024-12|DW46|
Scopula rubraria|NZPWP026-12|DW48|
Scopula rubraria|NZPWP017-12|DW39|
Scopula rubraria|NZPWP069-12|DW91|
Scopula rubraria|NZPWP027-12|DW49|
Chloroclystis|NZPWP114-13|PW020|
Chloroclystis|NZPWP115-13|PW021|
Chloroclystis|NZPWP242-13|PW158|

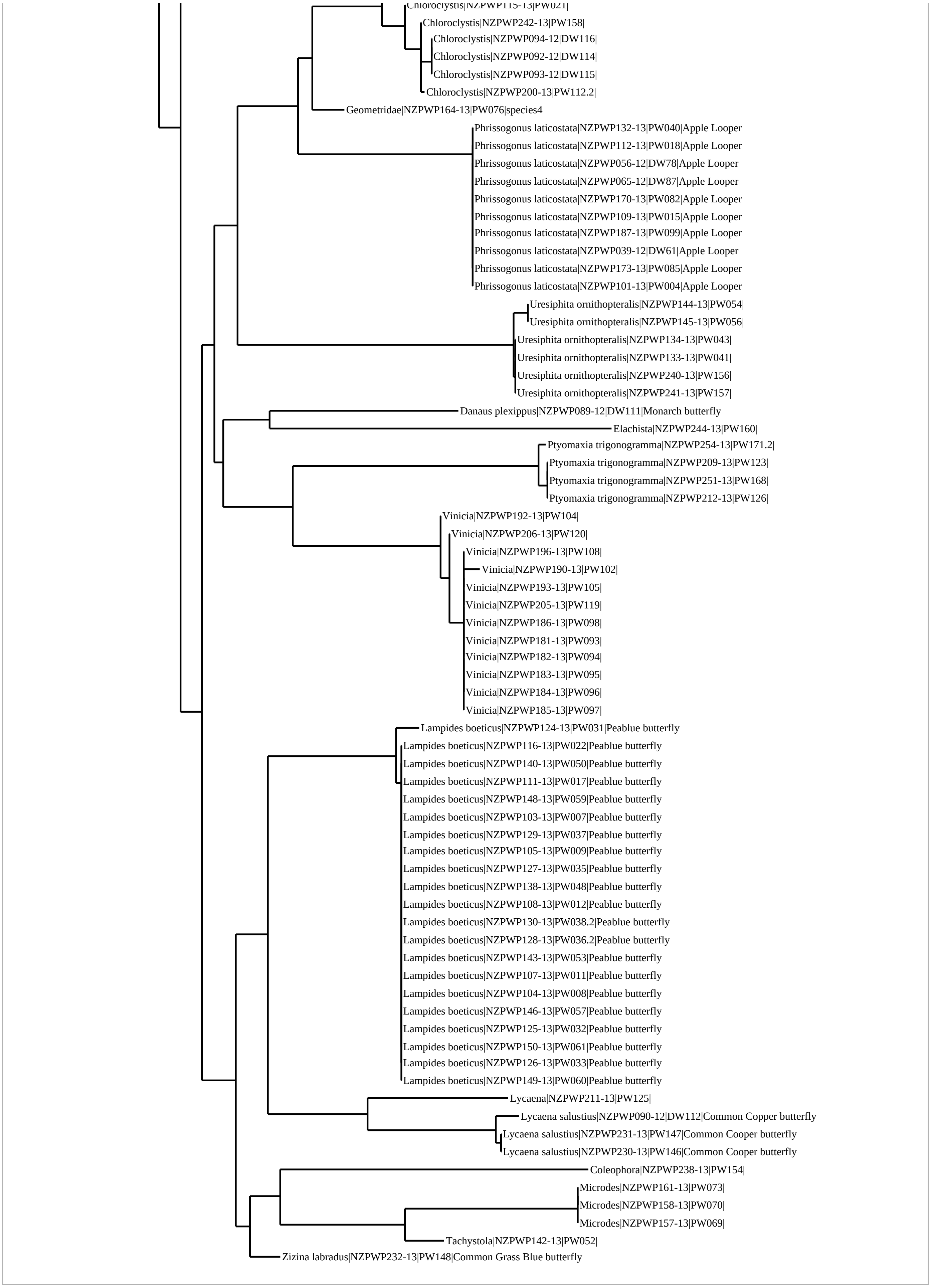
Chloroclystis|NZPWP115-13|PW021|
Chloroclystis|NZPWP242-13|PW158|
Chloroclystis|NZPWP094-12|DW116|
Chloroclystis|NZPWP092-12|DW114|
Chloroclystis|NZPWP093-12|DW115|
Chloroclystis|NZPWP200-13|PW112.2|
Geometridae|NZPWP164-13|PW076|species4
Phrissogonus laticostata|NZPWP132-13|PW040|Apple Looper
Phrissogonus laticostata|NZPWP112-13|PW018|Apple Looper
Phrissogonus laticostata|NZPWP056-12|DW78|Apple Looper
Phrissogonus laticostata|NZPWP065-12|DW87|Apple Looper
Phrissogonus laticostata|NZPWP170-13|PW082|Apple Looper
Phrissogonus laticostata|NZPWP109-13|PW015|Apple Looper
Phrissogonus laticostata|NZPWP187-13|PW099|Apple Looper
Phrissogonus laticostata|NZPWP039-12|DW61|Apple Looper
Phrissogonus laticostata|NZPWP173-13|PW085|Apple Looper
Phrissogonus laticostata|NZPWP101-13|PW004|Apple Looper
Uresiphita ornithopteralis|NZPWP144-13|PW054|
Uresiphita ornithopteralis|NZPWP145-13|PW056|
Uresiphita ornithopteralis|NZPWP134-13|PW043|
Uresiphita ornithopteralis|NZPWP133-13|PW041|
Uresiphita ornithopteralis|NZPWP240-13|PW156|
Uresiphita ornithopteralis|NZPWP241-13|PW157|
Danaus plexippus|NZPWP089-12|DW111|Monarch butterfly
Elachista|NZPWP244-13|PW160|
Ptyomaxia trigonogramma|NZPWP254-13|PW171.2|
Ptyomaxia trigonogramma|NZPWP209-13|PW123|
Ptyomaxia trigonogramma|NZPWP251-13|PW168|
Ptyomaxia trigonogramma|NZPWP212-13|PW126|
Vinicia|NZPWP192-13|PW104|
Vinicia|NZPWP206-13|PW120|
Vinicia|NZPWP196-13|PW108|
Vinicia|NZPWP190-13|PW102|
Vinicia|NZPWP193-13|PW105|
Vinicia|NZPWP205-13|PW119|
Vinicia|NZPWP186-13|PW098|
Vinicia|NZPWP181-13|PW093|
Vinicia|NZPWP182-13|PW094|
Vinicia|NZPWP183-13|PW095|
Vinicia|NZPWP184-13|PW096|
Vinicia|NZPWP185-13|PW097|
Lampides boeticus|NZPWP124-13|PW031|Peablue butterfly
Lampides boeticus|NZPWP116-13|PW022|Peablue butterfly
Lampides boeticus|NZPWP140-13|PW050|Peablue butterfly
Lampides boeticus|NZPWP111-13|PW017|Peablue butterfly
Lampides boeticus|NZPWP148-13|PW059|Peablue butterfly
Lampides boeticus|NZPWP103-13|PW007|Peablue butterfly
Lampides boeticus|NZPWP129-13|PW037|Peablue butterfly
Lampides boeticus|NZPWP105-13|PW009|Peablue butterfly
Lampides boeticus|NZPWP127-13|PW035|Peablue butterfly
Lampides boeticus|NZPWP138-13|PW048|Peablue butterfly
Lampides boeticus|NZPWP108-13|PW012|Peablue butterfly
Lampides boeticus|NZPWP130-13|PW038.2|Peablue butterfly
Lampides boeticus|NZPWP128-13|PW036.2|Peablue butterfly
Lampides boeticus|NZPWP143-13|PW053|Peablue butterfly
Lampides boeticus|NZPWP107-13|PW011|Peablue butterfly
Lampides boeticus|NZPWP104-13|PW008|Peablue butterfly
Lampides boeticus|NZPWP146-13|PW057|Peablue butterfly
Lampides boeticus|NZPWP125-13|PW032|Peablue butterfly
Lampides boeticus|NZPWP150-13|PW061|Peablue butterfly
Lampides boeticus|NZPWP126-13|PW033|Peablue butterfly
Lampides boeticus|NZPWP149-13|PW060|Peablue butterfly
Lycaena|NZPWP211-13|PW125|
Lycaena salustius|NZPWP090-12|DW112|Common Copper butterfly
Lycaena salustius|NZPWP231-13|PW147|Common Cooper butterfly
Lycaena salustius|NZPWP230-13|PW146|Common Cooper butterfly
Coleophora|NZPWP238-13|PW154|
Microdes|NZPWP161-13|PW073|
Microdes|NZPWP158-13|PW070|
Microdes|NZPWP157-13|PW069|
Tachystola|NZPWP142-13|PW052|
Zizina labradus|NZPWP232-13|PW148|Common Grass Blue butterfly
